# Cytotoxicity of Various Endodontic Materials on Stem Cells of Human Apical Papilla

**DOI:** 10.7508/iej.2016.01.004

**Published:** 2015-12-24

**Authors:** Eshagh Ali Saberi, Hamed Karkehabadi, Narges Farhad Mollashahi

**Affiliations:** a * Oral and Dental Diseases Research Center and Department of Endodontics, Dental School, Zahedan University of Medical Sciences, Zahedan, Iran; *; b * Department of Endodontics, Dental School, Hamadan University of Medical Sciences, Hamadan, Iran*

**Keywords:** Biodentine, Calcium-Enriched Mixture, Cytotoxicity, Endodontic Biomaterials, Mineral Trioxide Aggregate, MTT Assay, Stem Cells of Apical Papilla

## Abstract

**Introduction:**

This *in vitro* study assessed and compared the cytotoxicity of mineral trioxide aggregate (MTA), calcium-enriched mixture (CEM) cement, Biodentine (BD) and octacalcium phosphate (OCP) on stem cells of the human apical papilla (SCAP).

**Methods and Materials:**

SCAPs were isolated from two semi-impacted third molars. The cells were cultured in wells of an insert 24-well plate and were then incubated. The plates were then removed from the incubator and randomly divided into four experimental groups that were exposed to 1-mm discs of set MTA, CEM, BD or OCP, and one untreated control group. After 24, 48 and 168 h, the plates were removed from the incubator and 3-[4, 5-dimethylthiazol-2-yl]-2, 5-diphenyl tetrazolium bromide (MTT) solution was added to each well. Data were analyzed at different time points using the repeated measures ANOVA followed by Bonferroni test and the level of significance was set at 0.05.

**Results:**

Cytotoxicity of the four materials was not significantly different from that of the control group at 24, 48 and 168 h (*P*>0.05). Two-by-two comparison revealed that cytotoxicity of MTA and CEM cement was significantly different from each other at 168 h (*P*<0.05) although the cytotoxicity of CEM was less than MTA. Cytotoxicity of OCP and MTA was also significantly different from each other at 48 h and OCP had more favorable biocompatibility than MTA (*P*<0.05).

**Conclusion:**

CEM, OCP, BD and MTA showed acceptable biocompatibility when exposed to SCAP. Over time, CEM showed the least cytotoxicity among the materials under study.

## Introduction

By introduction of new bioactive and bioregenerative materials, tremendous advances have been made in endodontics. Regenerative endodontic treatment of immature necrotic teeth using mesenchymal stem cells (MSCs) have shown high success rates [1-3]. Biomaterials must be able to stimulate MSCs to form hard tissue and induce their differentiation to odontoblast-like cells. These biomaterials are in direct contact with cells and therefore, their biocompatibility is a critical requirement [4]. 

Mineral Trioxide Aggregate (MTA) is among the most commonly used biomaterials in regenerative endodontic treatment. It has excellent biocompatibility and is supplied in the form of a powder with small hydrophilic particles that set in the presence of moisture [5]. Due to optimal properties such as hydrophilicity, suitable radiopacity, high pH, setting expansion, low solubility and biocompatibility, MTA is used as a standard biomaterial for various endodontic procedures such as vital pulp therapy, regeneration, repair of root perforations, apexification and root end filling [5-7]. It is not fully understood how MTA stimulates the odontoblastic differentiation in human dental pulp stem cells (HDPSC); calcium ions, however, seem to be a major role player in this phenomenon (8).

Biodentine (BD) is a new aggregate of the Portland cement family with improved physicochemical and biological properties [8, 9]. The mechanical properties of BD resemble those of dentine.

It is supplied in the form of a powder containing tricalcium silicate, dicalcium silicate, calcium carbonate and zirconium oxide as opacifier and a liquid containing calcium chloride in an aqueous solution in combination with polycarboxylate as a dispersant.

**Figure 1:**
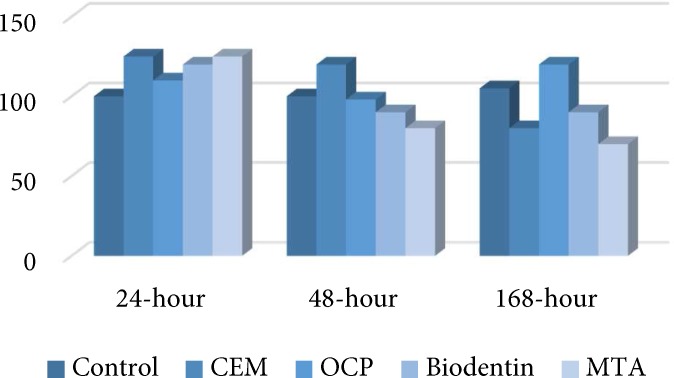
The percentage of cell viability in CEM, MTA, BD, OCP and the control groups after 24, 48 and 168 h

The powder is encapsulated and is mixed with the liquid in an amalgamator for 30 sec. It takes 12 min to set [10]. Since BD possess similar chemical composition to MTA, it is likely that the calcium ions released from BD are accountable for the survival of MSCs [10].

Calcium-enriched mixture (CEM) cement is another biomaterial containing different compositions of calcium. Its clinical applications are similar to those of MTA. CEM releases calcium hydroxide during and after setting and has a shorter working time, higher flow and lower film thickness than MTA. It is also capable of forming HA using an internal source of ions [11-14], which is a natural product of dental pulp cells. This property, similar to the reaction explained in the case of MTA, might have a role in the biocompatibility of CEM [15] .

Octacalcium phosphate (OCP) is another biomaterial with confirmed odontoblastic and favorable pulp healing effects. It is a calcium phosphate cement and has been suggested to be a direct precursor of hydroxyapatite (HA). It is capable of inducing the differentiation of dental pulp stem cells to odontoblast-like cells [16]. In comparison with other calcium phosphate cements, OCP has higher potential for induction and stimulation of hard tissue formation. In the process of new hard tissue formation, OCP is absorbed overtime and replaced with the newly formed hard tissue [17].

The cytotoxic effects of the above-mentioned endodontic biomaterials on gingival and periodontal fibroblasts, dental pulp stem cells and stem cells of the human apical papilla (SCAPs) have been confirmed in several previous studies [8, 18, 19]. Many *in vitro* studies proved the biocompatibility of these biomaterials and their ability to induce odontoblast and osteoblast differentiation and biomineralization in MSCs [6, 8, 10, 19, 20].

Considering the gap of information and limited number of studies on the biocompatibility of endodontic biomaterials exposed to SCAP, the aim of this *in vitro* study was to compare the cytotoxicity of MTA, CEM, OCP and BD on human SCAP using tetrazolium bromide (MTT) assay. 

## Materials and Methods

***Cell culture***

Stem cells were isolated from two immature mandibular third molars. Immediately after extraction, the teeth were rinsed and stored in sterile phosphate buffered saline (PBS) solution (Gibco BRI, Grand Island, NY, USA). Stem cells were then isolated from the tooth apical papilla by enzymatic digestion using type I collagenase (2 mg/mL) (Worthington Biomedical, Lakewood, NJ, USA) and immersed in Dulbecco’s modified Eagle’s medium suspension (DMEM, Gibco, Grand Island, NY, USA). To obtain higher number of cells, they were cultured again in a culture medium containing 15% fetal bovine serum (FBS) (Gibco BRI, Grand Island, NY, USA). This cell line was cultured in sterile culture medium containing 10% bovine serum (DMEM) in sterile cell culture flasks (SPL Life Science, Gyeonggi-do, South Korea). The culture medium was refreshed every 2-3 days during the process of cell culture and the cells were passaged after one week. After 4 passages, cells reached adequate confluence for cytotoxicity testing. 

Insert 4.0-μm plates (SPL Life Science, Gyeonggi-do, South Korea) were used for cell treatment. To assess the cytotoxic effects of the understudy materials, 20000 cells were cultured in each well of an insert 24-well plate. These plates enable indirect contact between the materials and cells to prevent cell lysis. Cells were incubated at 37^°^C for 24 h under 5% CO_2_ and 95% humidity (Binder, NY, USA). Plates were then removed from the incubator and the medium was changed. Next, SCAP were randomly divided into 4 experimental groups and 1 mg of the understudy biomaterials namely ProRoot MTA (Dentsply Tulsa Dental Specialties, Tulsa, OK, USA), Biodentine (Septodont, Saint-Maur-des-Fosses, France), CEM cement (BioniqueDent, Tehran, Iran) and OCP, under sterile laboratory conditions were prepared in the form of discs according to the manufacturers’ instructions and placed in the wells. The cells cultured in DMEM were used as a control group. Plates were removed from the incubator at 24, 48 and 168 h and 10 mL of MTT solution (Sigma Aldrich, St. Louis, MO, USA) and 90 mL of the DMEM culture medium containing 10% FBS were added to plates and they were incubated at 37^°^C for 4 h under 95% humidity and 5% CO_2_. The overlaying culture medium in each well was gently extracted and 100 mL of dimethyl sulfoxide (DMSO) (Gibco BRL, Grand Island, NY, USA) was added. After dissolution of formazan crystals, the optical density (OD) of the solution was read at 540 to 690 nm wavelengths using microplate reader (ELX808 absorbance microplate reader; BioTek Instruments Inc., Winooski, VT, USA). 

Data were analyzed with GraphPad Prism software (GraphPad Software, San Diego, CA, USA) using the repeated measures ANOVA for analysis at different time points followed by the Bonferroni test. The level of significance was set at 0.05. 

## Results

After 24, 48 and 128 h, there was not any significant differences between either of the tested biomaterials and the negative control group (*P*>0.05). Also, the percentage of viable cells slightly increased after exposure to biomaterials after 24 h and this increase was minimal in the CEM group (Figure 1). After 48 h the highest and lowest cell viability belonged to the OCP and MTA, respectively. Cell viability followed a decreasing pattern after 48 h compared to 24-h results. After 48 h, cell viability in the MTA group was significantly different from OCP group (*P*<0.01). The percentage of viable cells exposed to MTA was much lower compared to CEM cement (Figure 1).

After 168 h, the cell viability in all groups was lower than the control samples except for CEM cement. Cell viability in all groups decreased after 168 h, except for CEM group, which showed an ascending trend. At 168 h, cell viability in the MTA group was significantly different from that of CEM group (*P*<0.05) and the percentage of viable cells after exposure to CEM was higher compared to MTA (Figure 1). 

Inter-group comparison revealed that cytotoxicity of MTA was significantly different after 24 and 48 h (*P*<0.01) and also after 24 and 168 h (*P*<0.01). In MTA group, proliferation of stem cells exposed to MTA had a descending trend over time. The cytotoxicity of BD was not significantly different at 24 and 48 h or at 48 and 168 h (*P*>0.05). However, the cytotoxicity of BD was significantly different at 24 and 168 h (*P*<0.05) and proliferation of cells exposed to BD had a descending trend over time. The cytotoxicity of OCP was not significantly different at 24, 48 and 168 h (*P*>0.05). Although the proliferation of stem cells exposed to OCP had a descending trend, this decrease was not statistically significant. The cytotoxicity of CEM cement was not significantly different either at 24, 48 and 168 h (*P*>0.05). Proliferation of stem cells exposed to CEM showed a descending trend at first followed by an ascending trend. However, none of these changes reached statistical significance.

During the first 24 h following exposure, number of viable cells notably increased in all test groups except for the CEM group, which showed a slight increase. At 48 h, number of viable cells decreased in all groups and the greatest changes were observed in the MTA followed by the BD groups; the least changes were noted in OCP and CEM groups. During 168 h, number of viable cells in MTA and BD groups did not change significantly but the percentage of viable cells increased in the CEM group and decreased in OCP group.

## Discussion

This study assessed the cytotoxicity of BD, MTA, CEM and OCP on human SCAP at different time points. After 24 h, the cytotoxicity of test materials in descending pattern was CEM>BD>MTA>OPC. At 48 h, the cytotoxicity followed this pattern: MTA>BD>CEM>OCP. After 168 h, the cytotoxicity was as follows: MTA>OCP>BD>CEM. 

Recent evidence indicates that necrotic immature teeth show a predictable response to regenerative treatments [21, 22]. Regenerative treatments are based on biological mechanisms and aim to replace the lost or damaged tissues such as dentin and root structures in addition to cells of pulp-dentin complex [21, 22]. Biocompatible materials are the essential of regenerative treatments and preserve the capacity and the potential of stem cells for proliferation, survival and repair [23] 

At least 5 different types of MSCs can differentiate into odontoblast-like cells naming dental pulp stem cells (DPSCs), stem cells of the human exfoliated deciduous teeth (SHED), SCAP, dental follicle progenitor cells (DFPC) and bone marrow-derived mesenchymal stem cells (BMMSC) [24-26]. The cell line used in this study was SCAP which was isolated from immature third molars that were within the process of root formation. These teeth provide an excellent source of cells required for regenerative treatments and their proliferation rate is three times more than that of dental pulp stem cells (DPSCs) [27]. On the other hand, SCAP have the ability to express CD_24_ marker (pluripotency) and DSPP (dentin sialoprotein) genes, indicating the potential of these cells for use in regenerative treatments [28, 29]. Due to the anatomical position of the SCAP, assessment of the behavior of these cells and the effect of biomaterials on them is extremely important.

Several methods are used for cytotoxicity testing among which, the MTT assay is the most commonly used test for assessing the cytotoxicity of materials against different cell lines including the gingival fibroblasts, DPSCs, SCAP, and the periodontal ligament fibroblasts [8, 18, 19, 30]. This assay is a standard ISO-approved method for assessment of cytotoxicity [8]. MTT assay, *aka* Mossman’s Tetrazolium Toxicity assay, is a colorimetric assay for assessing cell viability. MTT, a yellow tetrazole, is absorbed by the mitochondria where it is reduced to purple formazan by succinate dehydrogenase in living cells. An acidified solution is added to dissolve the insoluble purple formazan product into a colored solution. The absorbance (optical density; OD) of this colored solution can be quantified by its measurement at a certain wavelength. By increased reduction of formazan and measurement of OD, cell viability and the cytotoxicity of materials can be measured.

MTA is the gold standard, calcium silicate-based, bioceramic cement and its biocompatibility has been confirmed in many *in vivo* and *in vitro* studies [4, 31, 32]. In the current study, no difference was found in the biocompatibility of MTA and BD. This result was in accordance with the findings of Chang *et al. *[33] who showed that BD had biocompatibility, inflammatory response and odontoblastic differentiation similar to those of MTA when tested on DPSCs. However, BD had physicomechanical properties superior to those of MTA and is similar to dentin in this regard. Moreover, BD has easier handling and shorter setting time than MTA. One study showed that MTA and BD induced the proliferation of different types of pulp cells [20] and this indicates their optimal biocompatibility. Calcium hydroxide is the final product after setting of MTA which plays a fundamental role in cell migration and proliferation and promotes cell survival at all concentrations [34]. Calcium chloride enhances the hydration reaction of MTA and plays the same role in BD liquid. This compound is hygroscopic and releases calcium ions [35].

In the current study, MTA and CEM did not show significant differences in the first 24 and 48 h; however, a significant difference was noted between them at 168 h (*P*<0.05); After 168 h, MTA and CEM cement showed the highest and the lowest cytotoxicity, respectively. Ghoddusi *et al.* [36] reported similar cytotoxic effects of CEM and MTA on mouse fibroblasts after 24, 48 and 72 h. However, Mozayeni *et al.* [12] reported higher cytotoxicity of CEM cement compared to MTA at 1, 7 and 24 days. Asgary *et al.* [13] showed that CEM cement and MTA had similar cell proliferation effects on DPSCs. Some other studies have also shown than CEM cement, similar to MTA, has optimal biocompatibility [12, 18, 37]. 

Such controversy in results may be related to the type of target cells, method of cytotoxicity assessment, direct contact of cells with the materials, concentration of materials and assessment time points. During the setting of calcium silicate-based cements, calcium silicate hydrate forms continuously and calcium carbonate phosphate deposits. Also, release of calcium ions can cause inflammatory toxic reactions [38]. On the other hand, release of this ion from silicate cements is important for the survival of MSCs [39]. This ion has signaling ability and plays an important role in up-regulation of cell functions. Migration of MSCs, BMSCs and tumoral cells is also influenced by calcium ions [40, 41]. Calcium silicate-based cements such as CEM cement and MTA are not an exception to this rule either. Difference in the percentage of cell viability in CEM cement and MTA groups at different time points in our study may be due to the different release of calcium ions. 

The chemical composition of MTA is different from that of CEM cement. Over time, the amounts of bismuth and silica leaching out from MTA increase while the release of calcium ion decreases. It has been documented that bismuth oxide does not encourage cell growth or proliferation [35]. In our study, comparison of MTA and OCP in the first 48 h revealed no significant difference. At 48 h following the exposure of stem cells to OCP, the highest cell proliferation was observed in this group, which was followed by a reduction in cell proliferation. It seems that due to the high proliferation of cells in the first 48 h and the resultant high cellular density and decreased availability of cell surfaces, the cells enter a growth decline phase thereafter. Calcium phosphate cements such as OCP have high pH. The biocompatibility and osteoconductivity of calcium phosphate cements have been well confirmed [42, 43]. Release of OH^-^, Ca and PO_4_ ions is among the characteristics of calcium phosphate cements and the ratio of calcium to phosphate ions is 1.67. High release of calcium ions from these compounds results in significant differences in cell viability in the first 48 h. OCP in the current study was prepared using the heterogeneous deposition method described by Le Geros in 1985. Comparison of the efficacy of OCP and calcium hydroxide for direct pulp capping revealed that the hard tissue barrier formed over time in calcium hydroxide group did not provide a good seal compared to that in the OCP group due to porosities [16]. 

In our study, no significant difference was noted between the test and control groups in terms of cell viability, which indicates that OCP, MTA, CEM and BD had optimal biocompatibility for use in regenerative treatments. This finding is in line with the results of previous studies separately assessing the biocompatibility of these materials [13, 18, 20, 44]. Release of calcium ions [39, 45] and high pH were common among the four understudy materials. Calcium ions play an important role in up-regulation of cell activities and our results showed that all understudy biomaterials had optimal biocompatibility. It appears that the release of calcium ions is an influential factor on biocompatibility and further evaluation of this topic in future studies can greatly help in choosing a suitable biomaterial for endodontic regenerative treatments.

In the current study, cells were exposed to set biomaterials. However, these biomaterials are used in the freshly mixed form in the clinical setting and it should be noted that biomaterials have higher toxicity during their setting because of the release of some toxic agents. After completion of setting, they become more structurally stable and their initial cytotoxicity decreases [18]. However, due to the presence of numerous confounding factors in the oral environment, the results of this *in vitro* study cannot be generalized to the clinical setting and clinical trials must be performed in this respect. 

## Conclusion

Time had a significant effect on increasing the cytotoxicity of octacalcium phosphate, Biodentine, CEM cement and MTA; however the cytotoxicity of CEM decreased over time. The four understudy biomaterials showed optimal biocompatibility when exposed to SCAP. 
